# Photoreceptor Degeneration in Two Mouse Models for Congenital Stationary Night Blindness Type 2

**DOI:** 10.1371/journal.pone.0086769

**Published:** 2014-01-21

**Authors:** Hanna Regus-Leidig, Jenny Atorf, Andreas Feigenspan, Jan Kremers, Marion A. Maw, Johann Helmut Brandstätter

**Affiliations:** 1 Department of Biology, Animal Physiology, FAU Erlangen-Nuremberg, Erlangen, Germany; 2 Department of Ophthalmology, University Hospital Erlangen, Erlangen, Germany; 3 Department of Biochemistry, University of Otago, Dunedin, New Zealand; Oregon Health & Science University, United States of America

## Abstract

Light-dependent conductance changes of voltage-gated Ca_v_1.4 channels regulate neurotransmitter release at photoreceptor ribbon synapses. Mutations in the human *CACNA1F* gene encoding the α1F subunit of Ca_v_1.4 channels cause an incomplete form of X-linked congenital stationary night blindness (CSNB2). Many *CACNA1F* mutations are loss-of-function mutations resulting in non-functional Ca_v_1.4 channels, but some mutations alter the channels’ gating properties and, presumably, disturb Ca^2+^ influx at photoreceptor ribbon synapses. Notably, a *CACNA1F* mutation (I745T) was identified in a family with an uncommonly severe CSNB2-like phenotype, and, when expressed in a heterologous system, the mutation was shown to shift the voltage-dependence of channel activation, representing a gain-of-function. To gain insight into the pathomechanism that could explain the severity of this disorder, we generated a mouse model with the corresponding mutation in the murine *Cacna1f* gene (I756T) and compared it with a mouse model carrying a loss-of-function mutation (ΔEx14–17) in a longitudinal study up to eight months of age. In ΔEx14–17 mutants, the b-wave in the electroretinogram was absent, photoreceptor ribbon synapses were abnormal, and Ca^2+^ responses to depolarization of photoreceptor terminals were undetectable. In contrast, I756T mutants had a reduced scotopic b-wave, some intact rod ribbon synapses, and a strong, though abnormal, Ca^2+^ response to depolarization. Both mutants showed a progressive photoreceptor loss, but degeneration was more severe and significantly enhanced in the I756T mutants compared to the ΔEx14–17 mutants.

## Introduction

Congenital stationary night blindness (CSNB) is a group of genetically heterogeneous, non-progressive retinal disorders that are inherited in an autosomal dominant, autosomal recessive or X-linked recessive manner. CSNB patients have impaired night vision, myopia or hyperopia, reduced visual acuity, nystagmus, abnormal electroretinograms (ERG) and disturbed dark adaptation [1], [2]. The X-linked forms of CSNB are classified into the complete (CSNB1) and the incomplete form (CSNB2). This classification is based on functional differences in the ERG [3], and on genetic analyses revealing two separate loci responsible for X-linked CSNB1 or CSNB2, respectively. Whereas CSNB1 is linked to mutations of the *NYX* gene [4], [5], mutations of the *CACNA1F* gene have been shown to cause CSNB2 [6]–[8]. The *CACNA1F* gene encodes the α1F subunit of Ca_v_1.4, an L-type voltage-gated Ca^2+^ channel that is located in the membrane at the active zone of photoreceptor and bipolar cell ribbon synapses [9], [10]. Dependent on the photoreceptor or bipolar cell membrane potential, Ca_v_1.4 channels regulate graded release of glutamate from these synapses [11], [12].

Some years ago, a novel mutation in the *CACNA1F* gene – encoding an amino acid substitution of threonine for isoleucine at position 745 of the CACNA1F protein (I745T) – was identified in a New Zealand family [13]. Members of this family exhibited unusually severe CSNB2-like symptoms [13]. Biophysical analysis of the Ca_v_1.4 channel gating properties in a heterologous expression system indicated that the I745T mutation leads to a remarkable −34 mV shift in the voltage dependence of channel activation, accompanied by slower inactivation kinetics [14]. A shift of channel activity to more negative membrane potentials should result in increased Ca^2+^ influx into photoreceptor terminals compared to wild-type Ca_v_1.4 channels. Therefore, the I745T substitution can be considered a gain-of-function mutation. However, it is not known whether the gating properties observed in the heterologous expression system [14] mirror the *in vivo* situation.

To gain insight into the pathomechanism that could account for the severity of the CSNB2-like disorder in the New Zealand family, we generated a mutant mouse containing the equivalent amino acid substitution of threonine for isoleucine at position 756 in *Cacna1f* exon 17 (*Cacna1f*I756T). Retinae of these mice were functionally and structurally characterized and compared with retinae of *Cacna1f* loss-of-function (*Cacna1f*ΔEx14–17; [15]) and wild-type mice up to eight months of age. The altered activity of I756T mutant channels affected the viability of rod photoreceptors more severely than non-functional ΔEx14–17 mutant channels, despite the maintenance of some communication between rod photoreceptors with I756T mutant channels and second-order-neurons.

## Materials and Methods

### Ethics Statement

The experiments were performed in compliance with the guidelines for the welfare of experimental animals issued by the Federal Government of Germany and the University of Erlangen-Nuremberg. The animal experiments were approved and registered by the local authorities (Amt für Veterinärwesen der Stadt Erlangen; AZ: TS - 10/07 Lehrstuhl für Zoologie-Tierphysiologie). Mouse breeding was performed in the animal facilities of the University of Erlangen-Nuremberg according to European and German laws on experimental animal welfare (Tierschutzgesetz; AZ 820-8791.2.63).

### Animals

ΔEx14–17 and I756T mutants were generated by Dr. Marion Maw, and are available through the Jackson Laboratory (stock # 017761 and # 017762). Information about original generation of these mouse lines is available on the Jackson Laboratory website and in Specht et al., 2009 [15]. In this study, ΔEx14–17 mutants (male, ΔEx14–17/Y; female ΔEx14–17/ΔEx14–17, I756T mutants (male, I756T/Y; female I756T/I756T; this study), and wild-type littermates (male, +/Y; female, +/+) maintained on a 12/12-h light/dark cycle with light on at 6 a.m. and with food and water ad libitum were used.

### Light Microscopy

Preparation of retinal tissue and antibody incubation for light microscopic immunocytochemistry was performed as described previously [10], [16]. Briefly, the eyes were opened and retinae were immersion fixed in the eyecup for 15–30 minutes in 4% paraformaldehyde (PFA) in phosphate buffer (PB; 0.1 M, pH 7.4). Retinae were mounted in freezing medium (Reichert-Jung, Bensheim, Germany), and twelve µm thick vertical sections were cut with a cryostat (Leica CM3050 S, Leica, Wetzlar, Germany). Primary antibody incubation was carried out overnight at room temperature, and secondary antibody incubation for one hour. Labeled sections were examined with a Zeiss Axio Imager Z1 equipped with an ApoTome (Zeiss, Oberkochen, Germany). Images were taken with a 100× (1.3 oil, Plan-Neofluar) objective as stacks of multiple optical sections, and projections were calculated with AxioVision 4.8 software (Zeiss, Oberkochen). Images were adjusted for contrast and brightness using Adobe Photoshop CS (Adobe, San Jose, CA, USA). Evaluation of cone photoreceptor numbers was carried out on retinal slices from three age-matched animals per genotype at the age of two and eight months. Fluorescein- or Rhodamine-conjugated peanut agglutinin (PNA; 1∶500; Vector Laboratories, Burlingame, CA, USA) was used to label cone photoreceptors [17]. The number of cone photoreceptor outer segments in a stretch of 100 µm ONL was counted in nine images from three retinal slices per animal.

### TUNEL Assay

TUNEL assays were performed with the Fluorescein *In Situ* Cell Death Detection Kit (Roche, Mannheim, Germany) on 12 µm thick PFA-fixed retinal cryostat sections according to manufacturer instructions except for a prolonged incubation step with permeabilisation solution for five minutes at room temperature. For quantification, TUNEL-positive cells on six retinal slices from three animals per genotype and per age group were counted. The number of photoreceptors was determined by counting DAPI stained nuclei in a field of 10,000 µm^2^ ONL/slice. The ratio of TUNEL-positive photoreceptor nuclei to the total number of photoreceptors was determined.

### Antibodies

The following antibodies were used: Monoclonal mouse anti-Bassoon mab7f (1∶2,500; Stressgen, MI, USA), mouse anti-GFAP (1∶500; Synaptic Systems, Göttingen, Germany), mouse anti-PKCα (1∶20,000; BD Biosciences, Heidelberg, Germany), polyclonal rabbit anti-Cacna1f (Pep3; 1∶3,000; [15]), rabbit anti-ubMunc13-2 (1∶6,000; [18]), rabbit anti-Calbindin (1∶1,000; Swant, Marly, Switzerland), polyclonal guinea pig anti-Pclo 44a (1∶4,000; [19]), guinea pig anti-VGluT1 (1∶50,000; Millipore, Billerica, MA, USA). DAPI was used to stain nuclei (1∶20,000; Sigma, Taufkirchen, Germany).

### Electron Microscopy

For conventional electron microscopy, retinae were fixed in 4% PFA and 2.5% glutaraldehyde in PB (0.1 M, pH 7.4) for 2 hours at room temperature, followed by incubation in 2% osmiumtetroxide for 1.5 hours, and embedded in Epon resin. Ultrathin sections were examined and photographed with a Zeiss EM10 electron microscope (Zeiss, Oberkochen, Germany) and a Gatan SC1000 OriusTM CCD camera (GATAN, Munich, Germany) in combination with DIGITAL Micrograph 3.1 software (GATAN, Pleasanton, CA, USA). Images were adjusted for contrast and brightness using Adobe Photoshop CS (Adobe, San Jose, CA, USA).

### Electroretinography

ERGs were measured from 4–6 animals per group and genotype. The detailed procedure for measuring the ERG in mice has been described elsewhere [20]. Briefly, the animals were dark adapted overnight and all further handling was performed under deep red illumination. The mice were anesthetized by an intramuscular injection of 50 mg/kg ketamine (Ketavet®, Pfizer) and 10 mg/kg xylazine (Rompun® 2%, Bayer). A subcutaneous injection of saline solution (10 ml/kg, 0.9%) protected the mice from desiccation. The pupils were dilated with a drop of tropicamide (Mydriaticum Stulln®, 5 mg/ml, Pharma Stulln GmbH) and phenylephrin-hydrochloride (Neosynephrin POS® 5%, Ursapharm). To measure the ERG, the ground needle electrode was placed subcutaneously at the base of the tail, the reference needle electrodes were positioned subcutaneously next to the ears and the active contact lens electrodes (Mayo Corporation, Japan), internally covered with Corneregel® (Dr. Mann Pharma), were placed on the cornea of each eye. To deliver the stimuli, a Ganzfeld Bowl (Roland Consult Q450 SC) was used. Stimulation and data recording were controlled using the RetiPort system (Roland Consult). Initially, the dark adapted flash ERG was measured. The flash strength increased in eight steps (0.0002, 0.002, 0.0063, 0.02, 0.063, 0.2, 0.63 and 6.3 cd.s/m^2^) and, depending on flash strength, 8 to 12 flashes were averaged. Flash duration varied between 5 µs and 5 ms depending upon the required total energy. After five minutes adaptation to 25 cd/m^2^ steady background light, photopic flash ERG measurements were performed. Flashes of five strengths (0.063, 0.2, 0.63, 2 and 6.3 cd.s/m^2^) were superimposed on the background. At each flash strength, 20 responses were averaged. ERG signals were amplified 100,000 times, band-pass filtered between 1 and 300 Hz, and digitized with a sampling frequency of 2048 Hz.

### Ca^2+^ Imaging

For Ca^2+^ imaging experiments, vertical slices of wild-type, ΔEx14–17, and I756T mouse retinae were cut at 200 µm with a vibratome (Leica, Mannheim, Germany). Subsequently, slices were incubated for 30–60 min at 37°C in an atmosphere of 5% CO_2_/55% O_2_ with 1 µM Fluo-4 AM (Life Technologies, Grand Island, NY) and 0.5 µl pluronic acid (Life Technologies) in a solution containing (in mM): 117 NaCl, 3 KCl, 2 CaCl_2_, 1 MgCl_2_, 0.4 NaH_2_PO_4_, 25 NaHCO_3_, and 15 Glucose (pH 7.4). Following incubation, slices were washed twice and immersed in an extracellular solution containing (in mM): 132 NaCl, 5.4 KCl, 5 CaCl_2_, 1 MgCl_2_, 5 Hepes, and 10 glucose (ph 7.4). Photoreceptor terminals in the slice preparation were depolarized with solutions containing 48 mM and 150 mM KCl. Osmolarity of the 48 mM KCl solution was adjusted to 300 mosm/l. Both depolarizing solutions were applied to the preparation with a focal perfusion system (ALA Scientific Instruments, Farmingdale, NY) controlled by Patchmaster software (Heka, Lambrecht, Germany). Imaging data were acquired with Axiovision software (Zeiss, Jena, Germany) at frame rates ranging from 5 to 20 Hz. Data analysis was performed with custom-made scripts using the software packages Matlab (MathWorks, Natick, MA) and Origin (Microcal, Northampton, MA). Time constants were measured by fitting a 1^st^ order exponential function to the decaying phase of the imaging signal.

## Results

### Age-dependent Reduction of CACNA1F Immunoreactivity in Mutant Photoreceptors

**Figure 1:**
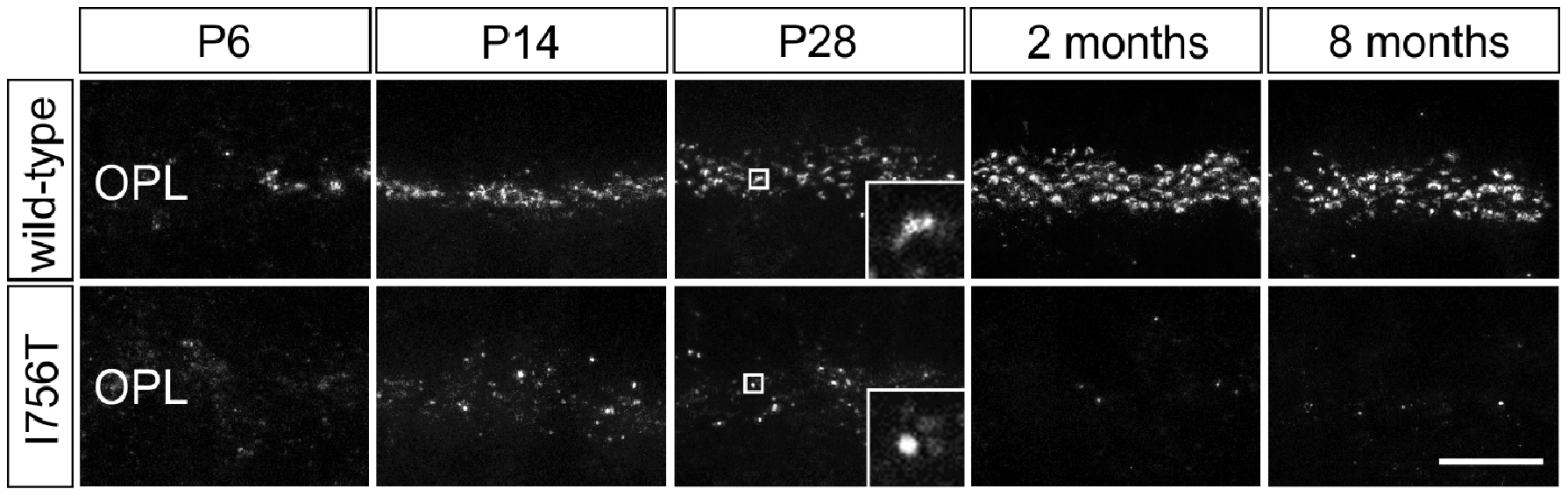
Age-dependent loss of CACNA1F immunoreactivity in the I756T mutant mouse. Immunocytochemical labeling of CACNA1F in wild-type and I756T mutant outer plexiform layer (OPL) at P6, P14, P28, two, and eight months. Scale bar: 10 µm.

In the first set of experiments, we examined the presence of CACNA1F in the outer plexiform layer (OPL) of I756T mutant retinae at postnatal day six (P6) to eight months of age using the Cacna1f(Pep3)-antibody [15] and compared the results with stainings of age-matched wild-type retinae (Fig. 1). In wild-type retina, CACNA1F immunoreactivity was already detectable in the developing OPL at P6. Labeling intensity increased from the time of eye opening (P14) until two months and decreased only slightly up to eight months, the latest time point examined (Fig. 1). In I756T mutant retinae, sparse CACNA1F immunoreactivity was detected at P14 and P28, but was almost absent by the age of two and eight months (Fig. 1). While CACNA1F immunoreactivity in wild-type retina showed the typical horseshoe shape of rod photoreceptor active zones (Fig. 1; P28 inset), in the I756T mutant it appeared as small discrete puncta (Fig. 1; P28 inset). In line with observations by Specht et al. [15], we detected residual CACNA1F protein in ΔEx14–17 mutant OPL at P28 and at two months, while at eight months CACNA1F was undetectable (not shown).

### Altered Morphology of Photoreceptor Ribbon Synapses in ΔEx14–17 and I756T Mutant Mice

**Figure 2:**
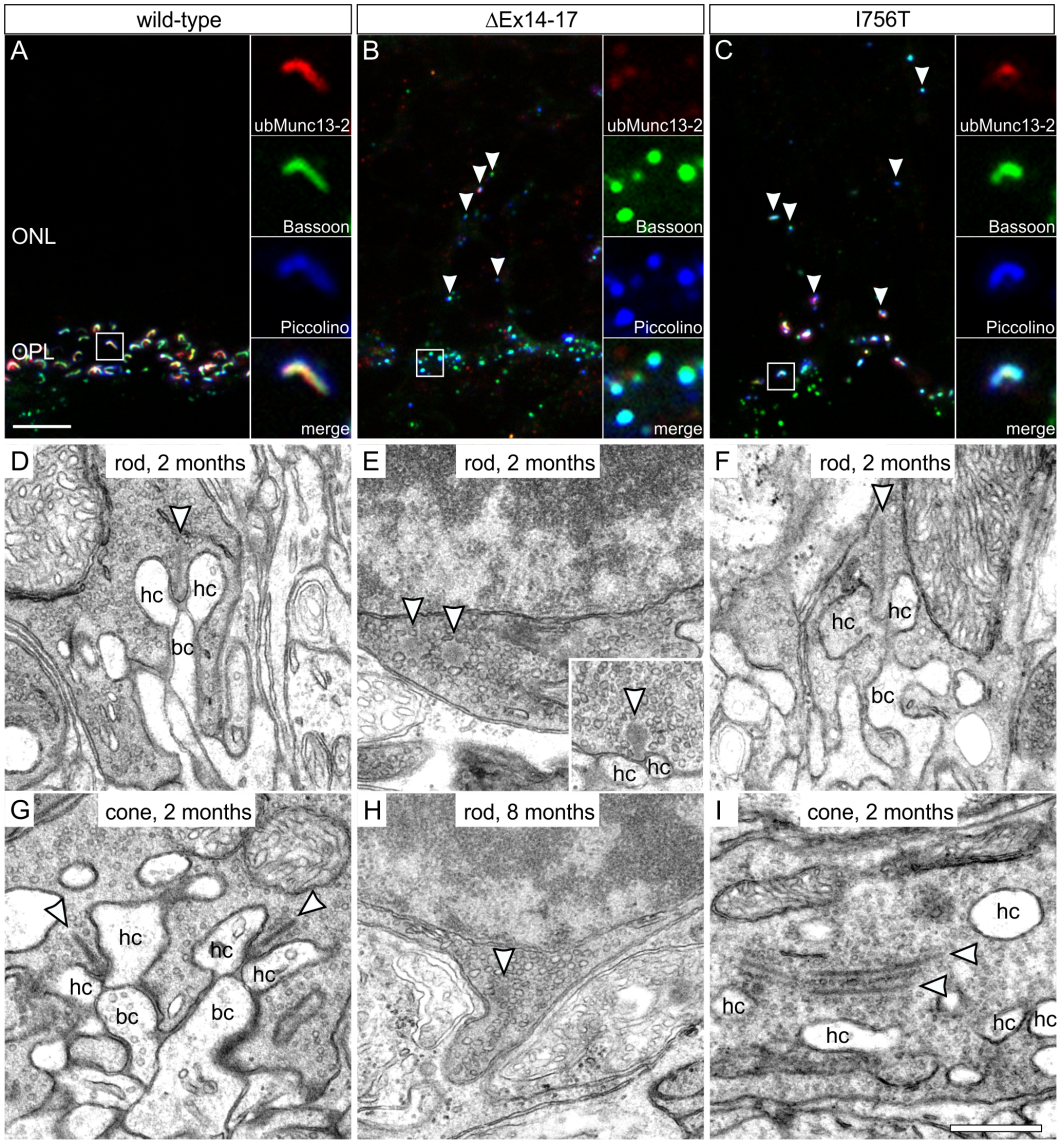
Photoreceptor ribbon synapse integrity in *Cacna1f* mutant mice. **A–C:** Immunocytochemical triple staining of ubMunc13-2 (red), Bassoon (green), and Piccolino (blue) on P28 old wild-type (A), ΔEx14–17 (B), and I756T (C) mutant outer retina. In ΔEx14–17 mutant rod photoreceptor terminals, ubMunc13-2 was nearly absent and colocalizing Bassoon/Piccolino-aggregates did not show the typical horseshoe shape. In I756T mutant rod photoreceptors, the horseshoe shaped co-localization of all three proteins indicates the presence of properly attached synaptic ribbons. Arrowheads point to ectopic synaptic sites in the ONL. **D–I:** Ultrastructural analysis of photoreceptor synapses in wild-type (D,G), ΔEx14–17 (E,H), and I756T (F,I) mutant retinae. Wild-type rod (D) and cone (G) photoreceptor synapses showed the typical plate-like synaptic ribbon (arrowhead) anchored to the presynaptic plasma membrane, and invaginating horizontal cell (hc) and bipolar cell (bc) processes at two months. In ΔEx14–17 mutant rod photoreceptors at two (E) and eight (H) months, ribbons (arrowhead) were spherically shaped and free-floating in most terminals, and postsynaptic invaginations were missing. Only rarely anchored ribbon material with facing postsynaptic elements could be observed (inset in E). In the two months old I756T mutant retina, remaining rod photoreceptor synapses (F) appeared largely intact, while many cone photoreceptor terminals (I) contained free-floating ribbons. ONL, outer nuclear layer; OPL, outer plexiform layer. Scale bar in A for A–C: 5 µm; in I for D–I: 0.5 µm.

To analyze the physical integrity of the photoreceptor synaptic ribbon at the arciform density, we triple labeled retinal cryostat sections of P28 wild-type, ΔEx14–17, and I756T mutant retina with antibodies against the arciform density protein ubMunc13-2 ([18]; red), the ribbon anchoring molecule Bassoon ([10], [16]; green), and the ribbon-specific protein Piccolino ([21]; blue) (Fig. 2A–C). In the wild-type OPL, the typical staining pattern in rod photoreceptor terminals shows a horseshoe-shaped Piccolino labeling bending around a similarly shaped Bassoon and ubMunc13-2-staining (Fig. 2A). In the ΔEx14–17 mutant OPL, immunoreactivity for Piccolino and Bassoon was punctate and co-localized in most cases, but ubMunc13-2 was almost undetectable (Figure 2B). Some fluorescent puncta were observed in the ΔEx14–17 mutant ONL, presumably representing ectopic synapses described in previous studies for this mouse line and other *Cacna1f* loss-of-function mutants (Figure 2B, arrowheads; [15], [22]–[24]). In the I756T mutant OPL, horseshoe shaped structures triple labeled for ubMunc13-2, Bassoon, and Piccolino were present, being a strong indication for anchored synaptic ribbons (Fig. 2C; [25], [26]). Synaptic staining was also detectable at ectopic sites in the I756T mutant ONL (Figure 2C, arrowheads; [25], [26]).

To verify the existence of morphologically intact photoreceptor ribbon synapses in the I756T mutant OPL, we performed an ultrastructural analysis of photoreceptor terminals and their ribbon synapses in two and eight months old wild-type, ΔEx14–17 and I756T mutant mice (Fig. 2D–I). In the wild-type, the typical ribbon synaptic configuration with a presynaptic ribbon facing three to four invaginating postsynaptic elements – two horizontal cell processes and one or two bipolar cell dendrites – could be seen in both rod and cone photoreceptor terminals (Fig. 2D,G). In line with the results from the *Cacna1f* G305X loss-of-function mutant analysis [27], and the observation by Zabouri and Haverkamp [24], in the ΔEx14–17 mutant retina, cone photoreceptor terminals were either absent or not identifiable as such. Furthermore, most rod photoreceptor terminals in the ΔEx14–17 mutant retina lacked invaginating postsynaptic elements, and synaptic ribbons were either absent or spherically shaped and free-floating at two and eight months (Fig. 2E,H). The rarely observed anchored rod photoreceptor ribbons were small and club-shaped (Fig. 2E; inset). In contrast to the malformed photoreceptor synapses of the ΔEx14–17 mutant, the morphology of the few remaining rod photoreceptor ribbon synapses in two months old I756T mutants appeared largely preserved (Fig. 2F). However, in I756T mutant cone photoreceptor terminals we observed free-floating ribbons at two (Fig. 2I) and at eight months (data not shown), which is comparable to the ribbon synaptic phenotype described for the Bassoon mutant mouse [16], [28]. Ultrastructural comparison of ectopic synapses in the ONL with synapses in the OPL of ΔEx14–17 and I756T mutant mice revealed no obvious structural differences.

### Altered ERGs in I756T and ΔEx14–17 Mutant Mice

**Figure 3:**
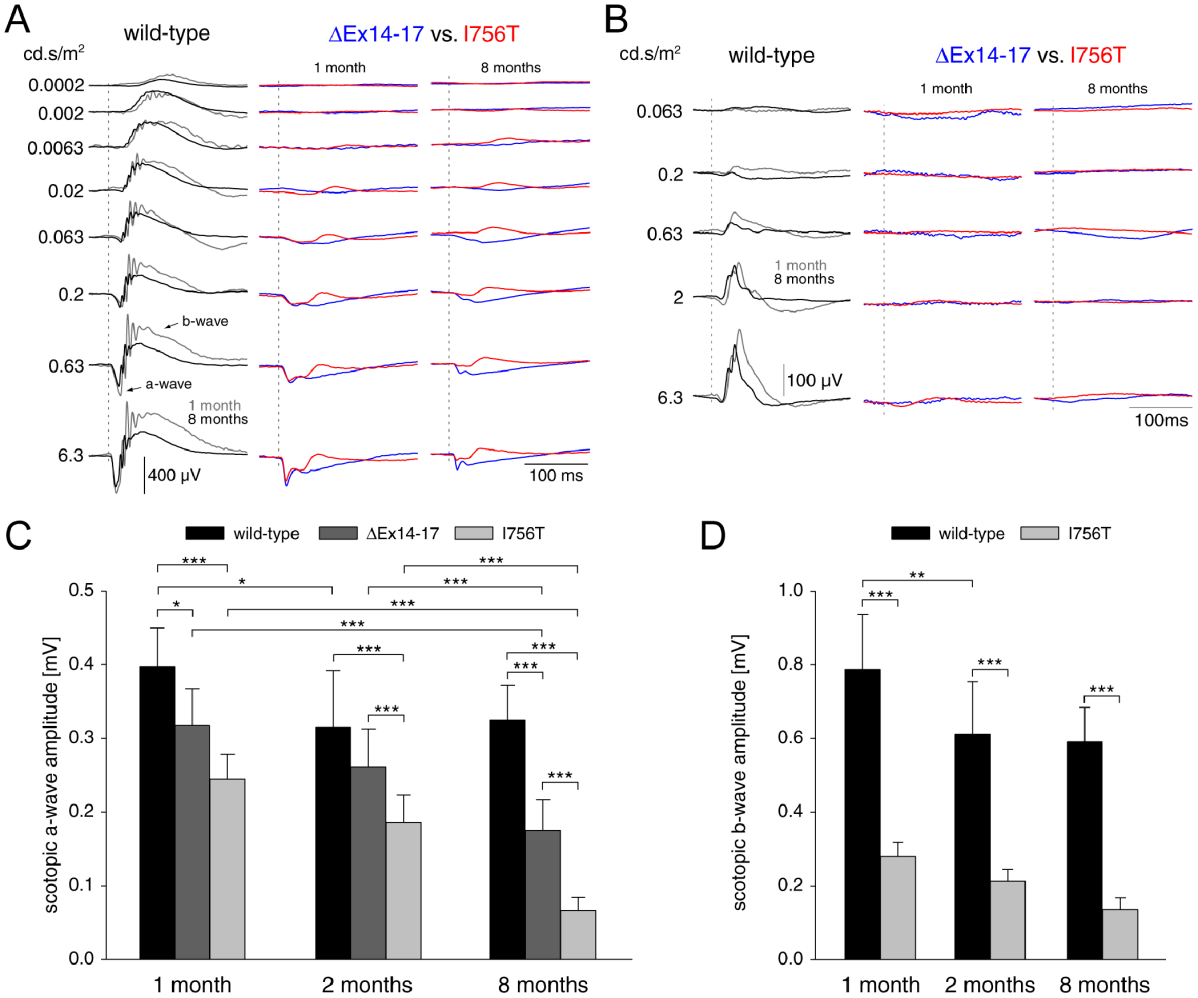
Flash ERG responses of *Cacna1f* mutant mice. **A:** Representative scotopic flash ERGs recorded to flashes of increasing strength in wild-type mice (left traces) at the age of one (grey traces) and eight (black traces) months. ERGs of ΔEx14–17 (blue traces) and I756T mutants (red traces) are separately compared at one (middle traces) and eight (right traces) months of age. In wild-type mice, the amplitude of the a- and b-wave increased with increasing flash strength and oscillatory potentials were visible on the rising part of the b-wave. The responses of both *Cacna1f* mutants were strongly altered in shape. Both mutants displayed a residual a-wave at higher flash strengths and I756T mutants also possessed a small b-wave. Vertical dashed lines indicate flash onset. **B:** Photopic flash ERGs in wild-type mice displayed a small a-wave and an increasing b-wave with increasing flash strength. In both *Cacna1f* mutants, no photopic flash ERGs were recordable, except of a small deflection at the highest measured flash strength in the responses of one month old I756T mutants. C,D: Age-dependent changes of the scotopic a- and b-wave amplitudes obtained at the highest flash strength of 6.3 cd.s/m^2^. **C:** In wild-type mice the a-wave amplitude decreased significantly (*p<0.5, ANOVA) between the age of one and 2 months and was stable thereafter. In *Cacna1f* mutants the a-wave amplitude decreased steadily with increasing age. Significant decreases are indicated by asterisks (**p<0.01, ***p<0.001, ANOVA). **D:** The b-wave amplitude of wild-type mice showed a significant decline between age one and 2 months (**p<0.01, ANOVA), but was stable thereafter. In the I756T mutants the residual b-wave decreased steadily in amplitude with increasing age.

To test retinal function, we performed ERG recordings of wild-type and *Cacna1f* mutant mice at the age of one, two and eight months. Figure 3A shows a summary of scotopic (Fig. 3A) and photopic (Fig. 3B) flash ERG responses to different flash strengths recorded in wild-type animals, in ΔEx14–17, and in I756T mutants. For the wild-type, a comparison of the ERGs is shown between the ages one and eight months, whereas the ERGs of the two mutants are compared separately for the two investigated ages. ERGs of another *Cacna1f* loss-of-function mutant [27] and of *nob2* mice, which carry a spontaneous *Cacna1f* null mutation [22], have been shown before and resemble those we recorded in the ΔEx14–17 mutants.

The scotopic flash ERGs of both *Cacna1f* mutants were strongly altered in comparison to wild-type animals (Fig. 3A). While the responses of ΔEx14–17 mutants only displayed a residual a-wave, the responses of I756T mutants also possessed a small b-wave. The response shape did not change with increasing age, but the response amplitudes decreased. At photopic conditions, flash ERGs were not recordable in ΔEx14–17 mutants and only a very small deflection could be measured in one month old I756T mutants at the highest measured flash strength.

Similar scotopic and photopic flash ERGs of I756T gain-of-function mutants have very recently been published by Liu et al. [25] and Knoflach et al. [26]. However, in their measurements only animals at five to six weeks of age were used. Here, we concentrated upon the age-dependent changes of the scotopic flash ERG between one and eight months. Figures 3C,D show the age-dependent changes of the scotopic a- and b-wave amplitudes at the highest flash strength of 6.3 cd.s/m^2^. In wild-type mice (Fig. 3C,D, black bars), the a- and b-waves displayed a small but significant reduction of the amplitude between the ages of one and two months (Fig. 3C: b-wave, p<0.05; Fig. 3D: a-wave, p<0.01, ANOVA) and did not change thereafter. The shape of the scotopic ERGs of two and eight months old ΔEx14–17 and I756T mutants resembled those at the age of one month, but the response amplitudes decreased with increasing age (Fig. 3A). The a-wave amplitudes of the mutants were significantly smaller compared to the wild-type at all ages, and, additionally, the a-waves measured in the I756T mutants were always smaller than those of ΔEx14–17 mutants. This difference was significant at the age of two and eight months (Fig. 3C: p<0.001, ANOVA). The amplitude of the residual b-wave measured in I756T mutants was significantly smaller in comparison to the wild-type at all ages. The residual b-wave amplitude showed a small and steady age-dependent decline (Fig. 3D). We did not observe any timing differences of the a- and b-waves in the mutant animals at any of the investigated ages.

### Enhanced Photoreceptor Degeneration in I756T Mutant Retina

**Figure 4:**
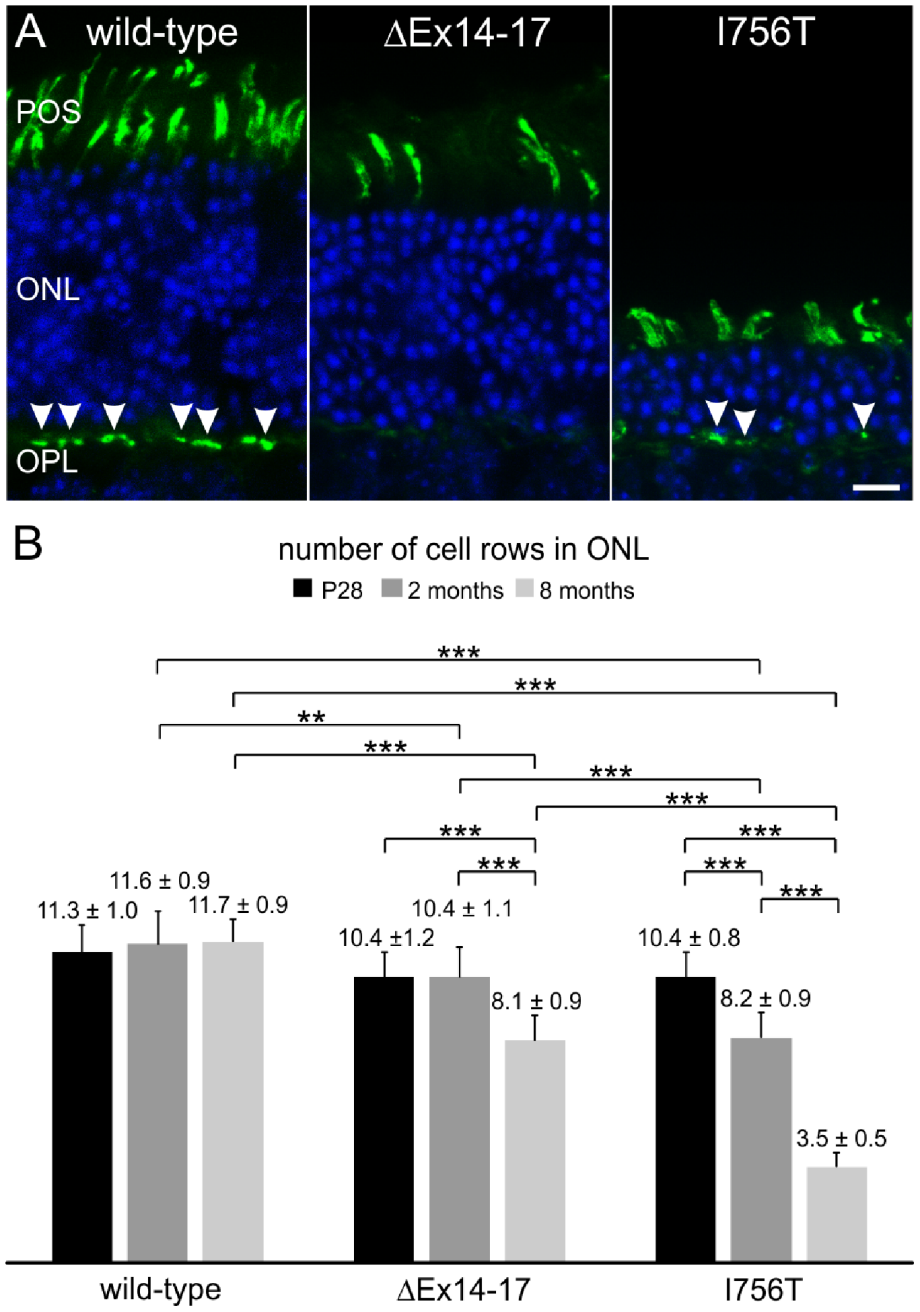
Age-dependent ONL thickness in *Cacna1f* mutant mice. **A:** Labeling of nuclei with DAPI (blue) and of cone photoreceptor outer segments and terminals (arrowheads) with peanut agglutinin (green) on retinal cryostat sections of wild-type, ΔEx14–17, and I756T mutant mice at 8 months. **B:** Quantification of the number of cell rows in the outer nuclear layer (ONL) from wild-type, ΔEx14–17, and I756T mutants at P28, 2 and 8 months. Values in are means ± SD. (*p<0.05; **p<0.01; ***p<0.001, ANOVA). POS, photoreceptor outer segments; OPL, outer plexiform layer. Scale bar: 10 µm.

To assess whether the age-dependent reduction of the scotopic a- and b-wave amplitudes and the drastic reduction of CACNA1F immunoreactivity in the OPL of aging *Cacna1f* mutant mice are correlated with progressing photoreceptor degeneration, we compared the outer retina of wild-type, ΔEx14–17 and I756T mutant mice at P28, and at two and eight months of age. Vertical cryostat sections were double labeled with the nuclear marker DAPI (Fig. 4A; blue) and the cone photoreceptor marker peanut agglutinin (PNA) (Fig. 4A; green), and the number of cell rows in the outer nuclear layer (ONL) was quantified (Fig. 4B). To avoid regional differences in retinal width, sections were taken from comparable locations with respect to the optic nerve. The analysis did not reveal any significant difference in the ONL thickness of the three genotypes at P28 (p>0.05; ANOVA), but a significant reduction in ONL thickness at two and eight months in both mutants compared to the wild-type (p<0.001; ANOVA). In both mutants, photoreceptor cell loss was more pronounced at eight months than at two months (p<0.001; ANOVA; Fig. 4A,B). Comparing the two mutants, in the I756T mutant retina photoreceptor loss was significantly more severe than in the ΔEx14–17 mutant at two and eight months, leading to a residual ONL thickness of only one-third of the wild-type retina at eight months (Fig. 4A,B; p<0.001; ANOVA).

In agreement with previous observations [25], the number of PNA labeled cone photoreceptor outer segments per 100 µm stretch of ONL were similar for the three genotypes at two months (wild-type: 13.7±2.1 cells; ΔEx14–17∶13.6±1.5 cells; I756T: 13.4±1.8 cells), At eight months, however, both *Cacna1f* mutants showed a significant reduction in the number of labeled cone photoreceptors compared to the wild-type (wild-type: 13.1±1.8 cells; ΔEx14–17∶8.7±2.5 cells; I756T: 9.5±1.6 cells; p<0.001; ANOVA). Furthermore, considering the moderate thinning of the ONL in the ΔEx14–17 mutant at eight months, the marked thinning of the ONL in the I756T mutant retina (Fig. 4B) suggests a preferential loss of rod photoreceptors. Despite PNA labeled cone photoreceptor outer segments remaining clearly visible in both *Cacna1f* mutants at eight months, labeling of their synaptic terminals persisted only in the I756T mutant retina (Fig. 4A; arrowheads). As demonstrated previously, in the ΔEx14–17 mutant retina the intensity of PNA labeled cone photoreceptor terminals was drastically reduced already at two months (not shown), and labeling was virtually absent at eight months (Fig. 4A; arrowheads; [24]).

**Figure 5:**
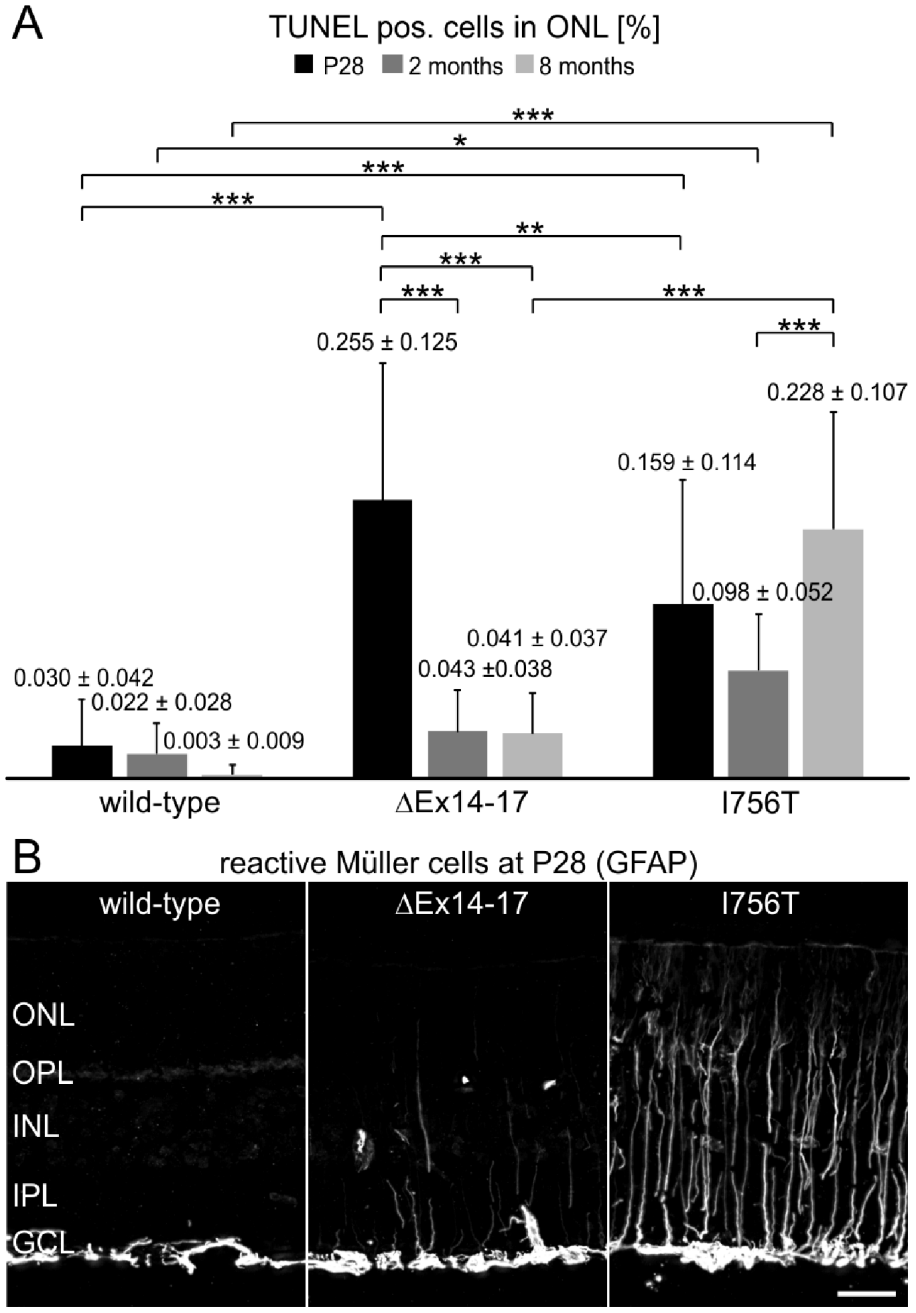
Photoreceptor degeneration in *Cacna1f* mutant mice. **A:** Quantification of the percentage of TUNEL positive cells in the ONL from wild-type, ΔEx14–17, and I756T mutants at P28, 2 and 8 months. Values are means ± SD. (*p<0.05; **p<0.01; ***p<0.001, ANOVA). **B:** Immunocytochemical staining of GFAP on P28 old wild-type, ΔEx14–17, and I756T mutant retina shows more pronounced Müller cell reactivity in the I756T mutant retina. ONL, outer nuclear layer; OPL, outer plexiform layer; INL, inner nuclear layer; IPL, inner plexiform layer; GCL, ganglion cell layer. Scale bar: 20 µm.

**Figure 6:**
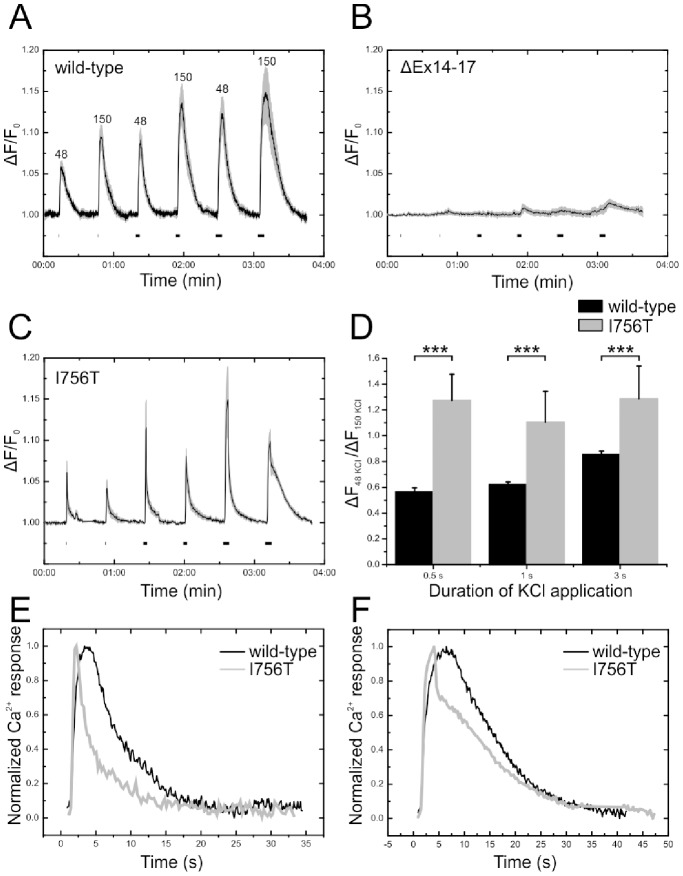
Photoreceptor Ca^2+^ responses are altered in *Cacna1f* mutant mice. **A–C:** 48^2+^ response measured as the change in fluorescence (ΔF/F_0_). The numbers above the peaks of the Ca^2+^ response in A show the respective KCl concentrations. In B and C numbers have been omitted for clarity. Horizontal bars below the traces indicate duration of KCl application (0.5 s, 1 s, 3 s). Plotted is the mean Ca^2+^ response of 8–10 sections (6 regions of interest each); the shaded area represents the s.e.m. **A:** Wild-type, **B:** ΔEx14–17, **C:** I756T retina. **D:** Bar graph summarizing the ratio of the Ca^2+^ response to 48 mM KCl and 150 mM KCl. Depicted is the mean ± s.e.m.; (***p<0.001; Kruskal-Wallis ANOVA). **E–F:** Normalized Ca^2+^ responses of wild-type and I756T mutant Ca_v_1.4 channels to 150 mM KCl for 0.5 s (E) and 3 s (F) at higher temporal resolution, displaying the differences in onset and decay kinetics.

To investigate the time course of photoreceptor degeneration, we performed TUNEL assays on cryostat sections of wild-type, ΔEx14–17, and I756T mutant retinae at P28, two and eight months (Fig. 5A). In the wild-type retina at P28 and two months, apoptotic photoreceptors accounted for only 0.030±0.042% and 0.022±0.028% of all photoreceptors and further dropped to 0.003±0.009% at eight months (Fig. 5A). In the ΔEx14–17 mutant retina at P28, the percentage of TUNEL positive cells in the ONL was 0.255±0.125%, a nine-fold increase compared to wild-type (p<0.001; ANOVA; Fig. 5A). At two and eight months, the percentage of TUNEL positive cells in the ΔEx14–17 mutant retina had decreased to 0.043±0.038% and 0.041±0.037%, respectively, which was not significantly different from the wild-type (p>0.05; ANOVA; Fig. 5A). This indicates an initial peak of photoreceptor degeneration in the young ΔEx14–17 mutant retina followed by a steady but moderate degeneration rate from two months onward. At all three time points, the I756T mutant retina showed a significantly increased rate of apoptosis compared to the wild-type (p<0.001; ANOVA; Fig. 5A). Given the declining rate of degeneration in the aging wild-type retina and the persistent rate of degeneration in the I756T mutant retina, there was a moderate four- to five-fold increase in apoptotic photoreceptors at P28 and two months (P28∶0.030±0.042% vs 0.159±0.114%; p<0.001; ANOVA; two months: 0.022±0.028% vs 0.098±0.052%; p<0.05; ANOVA; Fig. 6A), and a striking 70-fold increase at eight months (0.003±0.009% vs 0.228±0.107%, p<0.001; ANOVA; Fig. 5A). These results demonstrate an enhanced and comparatively steady rate of photoreceptor loss in the I756T mutant retina. The notion of enhanced photoreceptor degeneration in the I756T mutant retina is corroborated by a strong upregulation of GFAP in retinal Müller cells, as an indicator of retinal stress, already at P28 (Fig. 5B; [29]). In contrast, reactive Müller cells were absent in the wild-type, and only rarely observed in the ΔEx14–17 mutant retina at that age (Fig. 5B).

### Altered Gating Properties of I756T Mutant Ca_v_1.4 Channels in Mouse Photoreceptor Terminals

Unphysiological intracellular Ca^2+^ concentrations can trigger cell death pathways in sensory cells like photoreceptors [30], [31]. To judge whether voltage-dependent Ca^2+^ flux into the photoreceptor terminal is disturbed in the two *Cacna1f* mutants, we performed Ca^2+^ imaging experiments in retinal slice preparations from adult (P28) I756T and ΔEx14–17 mutant mice, and compared the results to Ca^2+^ responses obtained from adult wild-type mice (Fig. 6).

When characterized in a heterologous expression system, the voltage-dependence of Ca_v_1.4 channel activation peaked at ∼ 0 mV for wild-type channels whereas the mutant I745T channels peaked at ∼−30 mV [14]. According to the Nernst equation, extracellular concentrations of 48 mM and 150 mM KCl are required to depolarize the membrane to −30 mV and 0 mV, respectively (assuming an intracellular K^+^ concentration of 150 mM). 48 mM and 150 mM KCl were focally applied to Fluo-4 AM-loaded slices of the mouse retina for 0.5, 1, and 3 s while Ca^2+^ influx through voltage-gated Ca^2+^ channels was recorded. We waited for 30 s between applications to allow the Ca^2+^ signal to return to baseline. Ca^2+^ responses (F) were measured relative to a baseline image representing the average of the first ten images of the recording (F_0_), i.e. the ratio of fluorescence change (ΔF/F_0_) was determined.

Ca^2+^ responses increased with increasing duration of the depolarization in wild-type and I756T photoreceptor terminals (Fig. 6A,C). As expected for a loss-of-function mutation, Ca^2+^ responses in photoreceptor terminals with ΔEx14–17 mutant Ca_v_1.4 channels were barely detectable (Fig. 6B). For a given duration, depolarization of wild-type photoreceptor terminals with 48 mM KCl (≅ 30 mV) resulted in smaller Ca^2+^ response amplitudes in the photoreceptor terminals than depolarization with 150 mM KCl (≅ 0 mV; Fig. 6A). Therefore, the ratio of the response to 48 mM KCl (ΔF_48KCl_) and 150 mM KCl (ΔF_150KCl_) was consistently smaller than 1 in wild-type photoreceptor terminals (Fig. 6D).

In contrast to wild-type, in the I756T mutant, depolarization of photoreceptor terminals with 48 mM KCl (≅ 30 mV) caused consistently larger Ca^2+^ responses than depolarization with 150 mM KCl (≅ 0 mV), thus leading to a ΔF_48KCl/_ΔF_150KCl_ ratio larger than 1 (Fig. 6C,D). The differences in Ca^2+^ responses between wild-type and I756T mutant photoreceptor terminals were statistically significant (p<0.001; Kruskal-Wallis ANOVA), and demonstrate that the mutant channel retains the ability to regulate Ca^2+^ entry into the photoreceptor synaptic terminal in response to membrane depolarization, but that the voltage-dependence of this activity is dysfunctional. Such dysfunction is readily explained in terms of a shift of the activation curve of the I756T mutant channel towards more negative membrane potentials, as observed in the previous in vitro study of the I745T *CACNA1F* mutant. Such a voltage shift should result in pronounced activation of I756T mutant Ca_v_1.4 channels at relatively hyperpolarized membrane potentials; at membrane potentials that provide peak Ca^2+^ response amplitudes in wild-type Ca_v_1.4 channels, a significant fraction of I756T mutant Ca^2+^ channels should be inactivated. Of note, only ∼25% of I756T photoreceptor terminals displayed the significant increase in intracellular Ca^2+^ concentration, whereas most terminals did not respond to the KCl-induced depolarization at all. The latter were not considered for analysis, and they do not contribute to the mean in Figure 6C.

We also observed significant differences in the properties of the Ca^2+^ response of photoreceptor terminals between wild-type and I756T mutant Ca_v_1.4 channels when depolarized to 0 mV. The onset of the Ca^2+^ response measured as the time-to-peak amplitude was consistently faster in the mutant compared to wild-type Ca_v_1.4 channels (Fig. 6E,F). In addition, the time constant of the exponential decay (τ) was 3–5 times shorter in I756T mutant Ca_v_1.4 channels. However, this applied only to depolarizations lasting 0.5 s and 1 s, whereas 3 s depolarizations to 0 mV revealed a more complex decline of the mutant channel Ca^2+^ response with an initial fast decline that slowed after about 5 s (Fig. 6F). The analysis is summarized in Table 1.

**Table 1:** Ca^2+^ imaging of photoreceptor terminals.

Duration KClapplication (s)	KCl concentration(mM)	Wild-typeTime-to-peak (s)	*Cacna1f*I756TTime-to-peak (s)	Wild-type τ (s)	*Cacna1f*I756T τ (s)
0.5	48	1.81±0.24	0.62±0.05**	6.88±1.20	1.28±0.44**
	150	3.12±0.40	0.74±0.07**	5.18±0.69	1.05±0.09**
1	48	2.19±0.18	1.06±0.14**	3.99±0.28	1.79±0.44*
	150	3.87±0.51	1.03±0.10**	6.83±0.46	2.20±0.70**
3	48	3.92±0.14	3.34±0.39^n.s.^	5.86±0.67	2.27±0.74**
	150	4.47±0.55	3.45±0.68^n.s.^	12.91±1.19	14.04±1.59*

** p<0.01;

* p<0.05; n.s., not significant. Significance levels were determined by Kruskal-Wallis ANOVA.

In summary, the I756T mutant Ca_v_1.4 channels display altered gating properties not only in a heterologous expression system, but also in photoreceptor terminals as their physiological environment.

### Enhanced Sprouting of Rod Bipolar- and Horizontal Cell Processes into the ONL of the I756T Mutant

**Figure 7:**
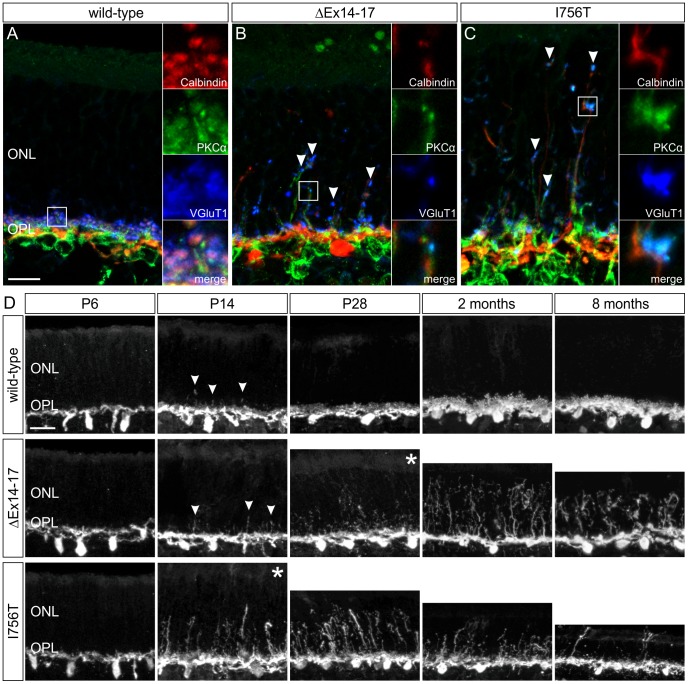
Comparison of the sprouting phenotype in the wild-type, ΔEx14–17, and I756T mutant retinae. **A–C:** Immunocytochemical triple staining of Calbindin (red), PKCα (green), and VGluT1 (blue) on P28 old wild-type (A), ΔEx14–17 (B), I756T (C) outer retinae shows sprouting of ON-bipolar cell dendrites as well as horizontal cell processes into the ONL of both *Cacna1f* mutants. The VGluT1 labeling shows the existence of presynaptic contacts with the sprouting elements. **D:** Comparison of the severity of horizontal cell sprouting in the wild-type, ΔEx14–17, I756T mutant outer retina at P6, P14, P28, two months, and eight months. Asterisks indicate the onset of noticeable sprouting in the *Cacna1f* mutants. In the I756T mutant retina, noticeable horizontal cell sprouting started earlier (P14) than in the ΔEx14–17 mutant retina (P28), but declined at eight months, when sprouting still continued in the ΔEx14–17 mutant retina. ONL, outer nuclear layer; OPL, outer plexiform layer. Scale bar in A for A–C**:** 10 µm; in D: 20 µm.

Photoreceptor synaptic plasticity and the formation of ectopic synaptic sites in the ONL of CSNB2 mouse models were described previously [15], [24]–[26]. Triple stainings confirmed the presence of sprouting of horizontal cell processes (Calbindin; red) as well as ON-bipolar cell dendrites (PKCα; green) into the ONL of both *Cacna1f* mutants, and VGluT1 labeling (blue) showed the existence of presynaptic contacts onto the sprouts (Fig. 7A–C). Liu et al. [26] reported that sprouting and ectopic synapse formation, indicating photoreceptor synaptopathies, was detected earlier in the ΔEx14–17 mutant ONL than in the I756T mutant ONL. We also analyzed the sprouting phenotype from P6 to 8 months of age by evaluating Calbindin-staining on retinal cryostat sections of wild-type, ΔEx14–17, and I756T mutant retina, and made a different observation (Fig. 7D). In all three genotypes, horizontal cell sprouts were not detected at P6. In wild-type retina, slight sprouting was observed at P14 but had disappeared by P28 (Fig. 7D; arrowheads). In ΔEx14–17 retina, a comparable slight sprouting was visible at P14 (Fig. 7D; arrowheads), which had increased at P28. We therefore defined P28 as the onset of increased sprouting in the ΔEx14–17 mutant retina (Fig. 7D; asterisk). Sprouting continued to increase at 2 month with slightly fewer sprouts at 8 months (Fig. 7D). By contrast, in the I756T mutant retina, a high number of elongated horizontal cell sprouts could already be observed at P14 (Fig. 7D; asterisk). Moreover, in I756T retina sprouting peaked at P28 and then declined with ONL sprouts observed only rarely at eight months (Fig. 7D).

## Discussion

Previous studies have implicated the Ca_v_1.4 channel in the formation and/or maintenance of photoreceptor synapses, and in mediating the graded release of neurotransmitter from photoreceptor terminals in response to illumination changes [11], [24], [26], [32]–[35]. In this study, we examined a mouse model generated for the I745T *CACNA1F* mutation found in a New Zealand family, which carries the isoleucine to threonine substitution at the equivalent position (I756T). This first *Cacna1f* gain-of-function model provides a new opportunity to probe the roles of the Ca_v_1.4 channel in health and disease.

### Functional Consequences of the I756T Cacna1f Mutation in Murine Photoreceptors

Immunolabeling with a CACNA1F antibody demonstrated that the I756T mutant protein is expressed and localized to photoreceptor ribbon synapses in the developing I756T mouse retina (Fig. 1). However, when compared to wild-type retina, there was only sparse labeling of ribbon synapses at P14 and P28. None-the-less, our Ca^2+^ imaging experiments in P28 retinal slice preparations of I756T mutant mice (Fig. 6) revealed that voltage-dependent Ca^2+^ flux is retained but abnormal in I756T photoreceptor synaptic terminals.

In darkness, wild-type photoreceptors are depolarized to ∼−40 mV, Ca_v_1.4 channels are activated and there is a high rate of glutamate release onto second-order neurons. With a bright light stimulus, photoreceptors hyperpolarize to ∼−65 mV, Ca_v_1.4 channel activity decreases and the rate of glutamate release declines correspondingly [11], [36]. In I756T mutant mice, electroretinography demonstrated a marked decrease in the amplitude, and increase of the delay, of the scotopic b-wave, implying impaired signal transmission from photoreceptors to second-order neurons (Fig. 3). The decreased amplitude of the scotopic b-wave may indicate a reduction in the dynamic range of the I756T mutant rod photoreceptors’ ability to transmit changes in illumination. Additionally, it may arise from photoreceptor degeneration and/or the occurrence of ectopic synapses.

Retention of rod signal transmission capability was consistent with the presence of ultrastructurally intact rod photoreceptor-to-bipolar cell synapses with anchored presynaptic ribbons and invaginating postsynaptic horizontal and bipolar cell processes at two and eight months of age (Fig. 2). The ultrastructure of cone photoreceptor terminals in the I756T mutant retina revealed free-floating ribbons (Fig. 2), and photopic b-wave responses were almost absent. Currently, we do not understand the structural and functional differences observed between rod and cone photoreceptor ribbon synapses in the I756T mutant retina.

### Comparison of Photoreceptor Degeneration and of Sprouting between the I756T Gain-of-function and ΔEx14–17 Loss-of-function Mutations

We observed a marked, progressive loss of rod photoreceptors in the I756T mutant retina (Fig. 4), accompanied by declining scotopic a-wave amplitudes (Fig. 3). Similar abnormalities occurred in the ΔEx14–17 mutant mice, albeit much less severely than in the I756T mutant mice (Figs. 3,4). The combination of staining with the cone photoreceptor marker PNA and quantification of photoreceptor cell rows in the ONL suggests that the putative loss of cone photoreceptors was milder than for rod photoreceptors in the I756T gain-of-function mutant, whereas in the ΔEx14–17 loss-of-function mutant, rod and cone photoreceptors seemed affected to a similar degree. These findings appear to be correlated with the ultrastructural abnormalities observed at the photoreceptor ribbon synapse: in those photoreceptors, which lacked invaginating postsynaptic elements and well-formed anchored ribbons, degeneration was slower than in those photoreceptors which retained those features (Figs. 2,4,5).

Sprouting of processes of second-order neurons into the ONL is a temporary phenomenon seen in wild-type retina during the period when photoreceptor ribbon synapses form and mature in the OPL ([37], this study). Sprouting and ectopic synaptogenesis is also found in many pathological conditions and may be regarded as a sign of photoreceptor dysfunction and degeneration [16], [34], [38], [39]. We observed sprouting of horizontal and bipolar cell dendrites into the ONL in both the ΔEx14–17 and I756T mutant retinae (Fig. 7). In these mice, this phenomenon might be regarded as a developmental dysfunction of synapse formation and maturation, and/or as an early symptom of retinal degeneration. However, this explanation seems to be inconsistent with the time courses of sprouting vs. measures of retinal degeneration such as ONL thinning and TUNEL positive cell counts. In ΔEx14–17 mutant retina, sprouting was highest at two and eight months, whereas TUNEL positive cells were highest at P28, i.e. the peak in cell death preceded, rather than followed, the peak in sprouting. Moreover, sprouting in I756T mutant retina declined in older mice, whereas TUNEL positive cells continued to be detected. In conclusion, these findings suggest that sprouting is poorly correlated with the time course of retinal degeneration.

Taken together, although both mutant mouse lines exhibited photoreceptor degeneration, sprouting and ectopic synapses, it was the I756 mutant that showed the more severe phenotype with respect to the extent of photoreceptor degeneration. As the ΔEx14–17 mutant lacks Ca^2+^ channel activity, and the I756T mutant not only retains but actually gains Ca^2+^ channel activity, we think that the increased Ca^2+^ concentration in the I765T mutant photoreceptor terminals is the trigger for the enhanced photoreceptor degeneration.

### Discrepancies between the Phenotypes of the Mouse Mutants and Human Patients

Unlike the retinal degeneration manifested by the I756T mouse mutant, affected individuals in the New Zealand family considered that their vision impairment was non-progressive [13]. Furthermore, for both children and middle-aged family members, their scotopic and photopic a-wave amplitudes were within 60–80% of amplitudes in control individuals ([13], Dianne Sharp, personal communication). Hyperpolarization of the photoreceptor in response to a light stimulus is thought to contribute to the a-wave. Accordingly, preservation of a-wave amplitudes makes it unlikely that significant photoreceptor degeneration occurs in the New Zealand family. However, there has not been long-term clinical investigation of family members such as regular electroretinography and optical coherence tomography to monitor potential changes in photoreceptor function and/or ONL thickness.

Again, unlike the ΔEx14–17 and the I756T mouse mutants, CSNB2 is generally considered to be a non-progressive disorder. However, a few *CACNA1F* mutations have been associated with variant progressive disorders, e.g. a splice site mutation in a family with cone-rod dystrophy [40], and an in-frame deletion and insertion in two brothers with retinal and optic disc atrophy [41].

The photopic b-wave phenotype of the I756T *Cacna1f* mutation in mice is also clearly different from that of the I745T *CACNA1F* mutation in human: the photopic b-wave is merely reduced, rather than absent, in affected family members [13]. This phenotypic discrepancy is, however, in line with similar findings for CSNB2 patients and *Cacna1f* loss-of-function mutants where the photopic b-wave response is also reduced in affected patients but absent in affected mice ([27], this study).

These phenotypical discrepancies between the human disorder and the mouse models might represent a species difference. One reason for the discrepancies may be the fact that the human life time is much longer and thus photoreceptor degeneration might have a later onset or might stay unnoticed for a long time. Alternatively or additionally, human photoreceptors might be equipped with more effective rescue mechanisms to substitute non- or malfunctioning proteins.

In conclusion, we have characterized a mouse model for a gain-of-function mutation in the *Cacna1f* gene. Our results suggest that synaptic ultrastructure and transmission from rod photoreceptors to second-order-neurons in retinae with non-functional Ca_v_1.4 channels is more impaired than in gain-of-function retinae. Conversely, rod photoreceptor degeneration in *Cacna1f* gain-of-function retina is more severe than in *Cacna1f* loss-of-function retina. While the mouse mutants therefore may not represent ideal models for the human disorders, future investigations of this novel gain-of-function model should provide new insights into the retinal roles of the Ca_v_1.4 channel.
